# Reduced hepatocellular lipid accumulation and energy metabolism in patients with long standing type 1 diabetes mellitus

**DOI:** 10.1038/s41598-019-39362-4

**Published:** 2019-02-22

**Authors:** Peter Wolf, Paul Fellinger, Lorenz Pfleger, Sabina Smajis, Hannes Beiglböck, Martin Gajdošík, Christian-Heinz Anderwald, Siegfried Trattnig, Anton Luger, Yvonne Winhofer, Martin Krššák, Michael Krebs

**Affiliations:** 1 Division of Endocrinology and Metabolism, Department of Internal Medicine III, Medical University of Vienna, Währinger Gürtel 18-20, 1090 Vienna, Austria; 2 Medical University of Vienna, Department of Biomedical Imaging and Image-guided Therapy, Centre of Excellence - High Field MR, Vienna, Austria

## Abstract

The prevalence of obesity and metabolic syndrome increases in patients with type 1 diabetes mellitus (T1DM). In the general population this is linked with ectopic lipid accumulation in liver (HCL) and skeletal muscle (IMCL), representing hallmarks in the development of insulin resistance. Moreover, hepatic mitochondrial activity is lower in newly diagnosed patients with T1DM. If this precedes later development of diabetes related fatty liver disease is currently not known. This study aims to investigate energy metabolism in liver (*k*_*ATP*_) and skeletal muscle (*k*_*CK*_) and its impact on HCL, IMCL, cardiac fat depots and heart function in 10 patients with long standing T1DM compared to 11 well-matched controls by ^31^P/^1^H magnetic resonance spectroscopy. HCL was almost 70% lower in T1DM compared to controls (6.9 ± 5% *vs* 2.1 ± 1.3%; p = 0.030). Also *k*_*ATP*_ was significantly reduced (0.33 ± 0.1 s^−1^ *vs* 0.17 ± 0.1 s^−1^; p = 0.018). In T1DM, dose of basal insulin strongly correlated with BMI (r = 0.676, p = 0.032) and HCL (r = 0.643, p = 0.045), but not with *k*_*ATP*_. In the whole cohort, HCL was significantly associated with BMI (r = 0.615, p = 0.005). In skeletal muscle *k*_*CK*_ was lower in patients with T1DM (0.25 ± 0.05 s^−1^ *vs* 0.31 ± 0–04 s^−1^; p = 0.039). No significant differences were found in IMCL. Cardiac fat depots as well as heart function were not different. Our results in patients with long standing T1DM show that HCL is lower compared to matched controls, despite reduced energy metabolism in liver and skeletal muscle.

## Introduction

The prevalence of obesity increases in patients suffering from type 1 diabetes mellitus (T1DM)^1^. Additionally, growing evidence suggests that liver and skeletal muscle are more insulin resistant in T1DM^2^. Obesity and insulin resistance represent two major features in the development of metabolic syndrome, which is associated with cardiovascular diseases and increased mortality in general population^3^.

Despite substantial reductions in the incidence of diabetes related comorbidities, including retinopathy and nephropathy, due to intensive diabetes treatment and better glycemic control^4^, overall mortality has remained substantially elevated even in patients with well controlled type 1 diabetes^5^. This increase in mortality is mainly due to cardiovascular diseases and is further enhanced with worsening of glycaemia^5^. Tight glycemic control in T1DM is mainly achieved by increasing daily insulin dose, which is closely linked with weight gain and thus might trigger metabolic disturbances^6^. Therefore, characterizing metabolic consequences of exogenous insulin therapy in patients with long standing T1DM is of utmost importance in order to detect future therapeutic targets to improve overall mortality.

In non-diabetic individuals, as well as in patients suffering from type 2 diabetes mellitus (T2DM), ectopic lipid deposition in liver and skeletal muscle represent hallmarks in the development of insulin resistance. However, data on the impact of ectopic fat accumulation for the development of insulin resistance in T1DM is conflicting. Whereas cross-sectional studies using ultrasound measurement techniques in 250 patients with T1DM suggest a prevalence of non-alcoholic fatty liver disease (NAFLD) up to 50%^7,8^, studies using proton magnetic resonance spectroscopy (^1^H MRS) showed a significantly lower hepatocellular lipid content (HCL) in obese patients with T1DM compared to non-diabetic control subjects^9,10^.

Alterations in mitochondrial activity might contribute to ectopic lipid accumulation in insulin sensitive tissues. Forward rates of skeletal muscle mitochondrial oxidative ATP turnover are reduced in insulin resistant individuals^11^ and correlate negatively with HCL^12^. Also, in the liver, insulin resistance relates to perturbed mitochondrial energy metabolism, since unidirectional flux through ATP synthase (F_ATP_) is lower in T2DM^13^. Interestingly, hepatic mitochondrial activity seems to adapt to HCL in early stages of NAFLD, since respiratory capacity is significantly increased compared to lean controls. However, this hepatic mitochondrial flexibility is subsequently lost when liver disease progresses^14,15^. Recent evidence in newly diagnosed patients with T1DM suggests lower hepatic ATP synthesis, independently of HCL^16^. If these alterations precede later diabetes-related liver disease and represent a risk to develop NAFLD in later life is not known yet.

With regard to the heart, cardiovascular disease is among the leading causes of death in T1DM. Whereas the overall rate of death from atherosclerosis and myocardial infarction constantly decreased during the last 10 years, no improvements were observed for death from heart failure^17^. In patients with T2DM excessive lipid accumulation within the myocardium (MYCL) is associated with diastolic dysfunction^18^. Moreover, not only MYCL, but also pericardial fat (PERI) might contribute to cardiac dysfunction directly by mechanical or paracrine effects^19^. In T1DM, only little is known on the impact of different cardiac fat depots on heart failure and alterations in cardiac geometry.

Therefore, the aim of this study was (i) to investigate tissue specific energy metabolism in insulin sensitive tissues in patients suffering from long-standing T1DM and (ii) to investigate its impact on ectopic lipid accumulation in liver, skeletal muscle and the heart compared to well matched healthy, non-diabetic controls.

## Methods

10 patients with long-standing T1DM were compared to 11 sedentary, healthy controls well matched for age, sex and body mass index (BMI) (Table 1). T1DM patients were required to be on stable dosage of functional insulin therapy for at least 3 months prior to screening visit. The amount of prandial insulin demands and therefore the actual daily absolute insulin dose was highly variable. Therefore only the dose of basal insulin was used for calculations.

**Table 1:** Baseline characteristics in patients with T1DM and controls; data are presented as mean ± standard deviation; GOT: glutamate – oxaloacetate - transaminase; GPT: glutamate –pyruvate – transaminase; GGT: gamma – glutamyl – transferase.

	T1DM	Controls	p-value
Sex (m/f)	6/4	6/5	n.s.
Age (years)	46 ± 5	50 ± 1	n.s.
BMI (kg/m^2^)	25.5 ± 2	26.8 ± 3	n.s.
T1DM duration (years)	25 ± 11		
Dose of basal insulin (IU/day)	21 ± 8.2		
Fasting glucose (mg/dl)	130 ± 44	92 ± 8	0.026
HbA1c (%)	7.4 ± 0.6	5.3 ± 0.3	<0.001
Creatinine (mg/dl)	0.80 ± 0.2	0.80 ± 0.14	n.s.
GOT (mg/dl)	28 ± 8	24 ± 7	n.s.
GPT (mg/dl)	27 ± 7	24 ± 7	n.s.
GGT (mg/dl)	19 ± 7	19 ± 14	n.s.
Triglycerides (mg/dl)	113 ± 21	149 ± 45	n.s.
Cholesterol (mg/dl)	180 ± 40	205 ± 36	n.s.
HDL–Cholesterol (mg/dl)	67 ± 20	60 ± 15	n.s.
LDL–Cholesterol (mg/dl)	90 ± 23	117 ± 34	0.049

Exclusion criteria were evidence of cardiovascular disease, hypoglycemia unawareness or manifest end-organ damage, i.e. diabetic nephropathy, neuropathy or retinopathy.

The ethical committee of the Medical University of Vienna, Austria approved performing the experiments. Written informed consent was obtained from all participating subjects. This study was conducted in full conformance with the relevant guidelines and regulations, i.e. principles of the Declaration of Helsinki and the ICH-GCP guidelines. The study was registered at www.clinicaltrials.gov (NCT02023489).

Subjects were investigated under resting conditions in the morning after an overnight fast of at least ten hours and were asked to refrain from intensive physical training, to stop regular moderate exercise and to ingest an isocaloric diet (30 kcal/kg/day, carbohydrate/protein/fat: 55%/15%/30%) for three days prior to the MR measurements. In T1DM group, stable blood glucose values and the absence of hypoglycemia prior to the study days had to be documented.

On the first study day, subjects underwent ^31^*P/*^1^*H magnetic resonance spectroscopy* (MRS) of the *liver and skeletal muscle*, which was performed on a 7 T MR System (Magnetom, Siemens Healthineers, Erlangen, Germany) using a double-tuned (^31 ^P/^1 ^H) surface coil (Rapid Biomedical, Rimpar, Germany), with a diameter of 10 cm. The liver measurements were performed in the right lateral position with the right lobe of the liver positioned over the coil. For estimating *hepatic mitochondrial activity (k*_*ATP*_*) by* ^31^*P MRS*, saturation transfer (ST) experiment was performed as described previously^14^. Concentrations of phosphor-containing hepatic metabolites were measured with 3D multi-voxel MRS based method for absolute quantification^20^. *Hepatocellular lipid content* was assessed with single-voxel ^1^*H MRS* using ultra-short echo time (TE = 6 ms) and calculated from ratios of summed area of methylene and methyl resonance to that of water following the individual T2 relaxation correction as per cent of total tissue MRS signal (water + methylene + methyl)^21^. The skeletal muscle measurements were performed in supine position with the calf muscle positioned over the same surface coil. Intramyocellular lipid content (IMCL) was measured in soleus muscle with single voxel ^1^*H MRS* using long echo time (TE = 280 ms) and calculated from ratios of area of IMCL methylene resonance to that of water following the individual relaxation correction as percentage of total tissue water MRS signal^22^. Unidirectional forward rates of oxidative ATP synthase (*k*_*ATP*_) and creatine-kinase (*k*_*CK*_) were measured in the same setting with the ^31^P channel of the surface coil applying saturation transfer technique as described previously^14^.

On another study day, ^1^*H MRI/MRS* of the myocardium was performed on a 3 T Tim Trio System (Siemens Healthineers, Erlangen, Germany). All healthy control subjects underwent both MR measurements. In the T1DM group seven patients participated at cardiac ^1^H MRI/MRS. *Myocardial lipid content (MYCL)* was measured by ECG-gated single voxel localized ^1^H MRS, as described previously^23^. For cardiac MR visualization, analysis of heart function and pericardial fat from T_1_-weighted CINE MRI, ARGUS software (Siemens) was used. For *pericardial fat (PERI)*, i.e. paracardial + epicardial adipose tissue, regions of interest were manually drawn along the borders of the fat surrounding the heart in three slices from the apex to the pulmonary trunk. The mean is given in square centimeters.

*Laboratory parameters* were measured by routine lab methods at AKH Vienna (http://www.kimcl.at).

Based on previous data^14,23,24^ a sample size of 8 subjects per group was sufficient to detect clinically relevant differences in ectopic lipid content (IMCL ± 0.8%, HCL ± 4%, MYCL ± 0.2%) with alpha = 0.05 and beta = 0.2. Exploratory statistical analysis was performed using SPSS Version 24 (IBM, Armonk, NY, USA). Data is given as means ± standard deviation. Comparison between groups was performed by unpaired Student’s t-test. Correlation analysis was calculated with Pearson’s correlation coefficient (r). Level of statistical significance was set at α < 0.05.

## Results

### Baseline characteristics

T1DM and controls were of comparable age and BMI. HbA1c and fasting blood glucose was significantly higher in T1DM. Anthropometric characteristics and laboratory values are summarized in Table 1.

### Hepatic energy- and lipid metabolism

**Figure 1:**
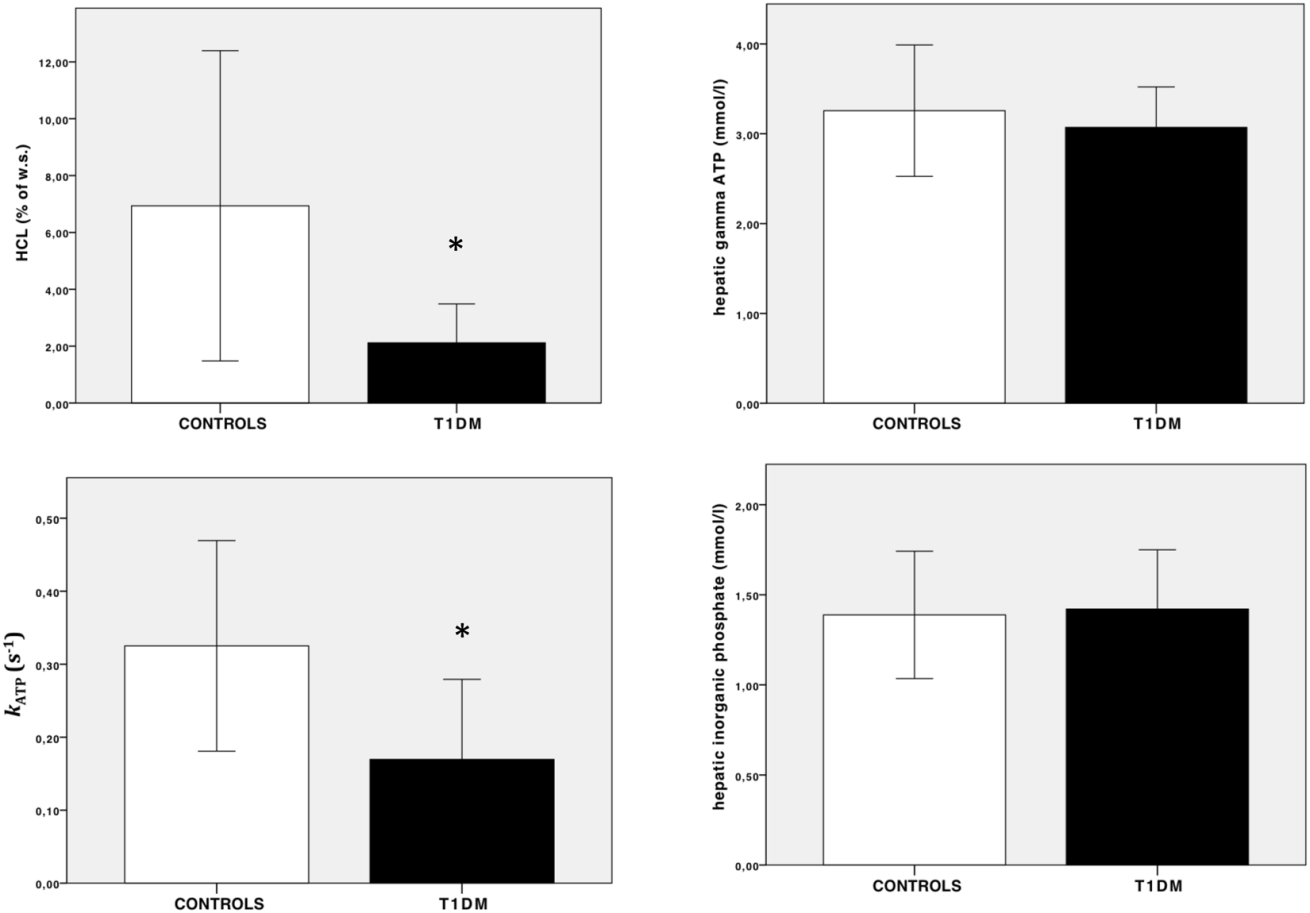
Hepatocellular lipid content (HCL), hepatic *k*_ATP_ and concentrations of *γ*-ATP & inorganic phosphate (Pi) in patients with T1DM and matched healthy controls. *Indicates p < 0.05; Data are presented as means ± SD.

HCL was almost 70% lower in T1DM compared to controls (controls *vs* T1DM: 6.9 ± 5% *vs* 2.1 ± 1.3%, p = 0.030). *k*_*ATP*_ was significantly reduced by half in T1DM (controls *vs* T1DM: 0.33 ± 0.1 s^−1^ *vs* 0.17 ± 0.1 s^−1^, p = 0.018) (Fig. 1). No differences could be found in absolute concentrations of energy rich phosphate compounds (controls *vs* T1DM: *γ*-ATP = 3.2 ± 0.7 mmol/l *vs* 3.0 ± 0.4 mmol/l, p = n.s.; inorganic phosphate = 1.4 ± 0.3 mmol/l *vs* 1.4 ± 0.3 mmol/l, p = n.s.; phosphatidylcholine = 1.0 ± 0.3 mmol/l *vs* 1.2 ± 0.4 mmol/l, p = n.s., phosophoethanolamine = 1.43 ± 0.6 mmol/l *vs* 1.55 ± 0.5 mmol/l, p = n.s., NADH = 1.1 ± 0.2 mmol/l *vs* 1.1 ± 0.2 mmol/l, p = n.s). Dose of basal insulin strongly correlated with BMI (r = 0.676, p = 0.032) and HCL (r = 0.643, p = 0.045), while no significant associations were observed for *k*_*ATP*_ and concentrations of phosphate compounds. In the whole cohort, HCL was significantly associated with BMI (r = 0.615, p = 0.005).

### Skeletal muscle energy- and lipid metabolism

No significant differences were found in soleus IMCL (0.50 ± 0.4% *vs* 0.88 ± 0.5%, p = 0.114). *k*_*CK*_ was markedly reduced in T1DM (0.25 ± 0.05 s^−1^ *vs* 0.31 ± 0.04 s^−1^, p = 0.039). No differences in the basal levels of phosphorus-containing metabolites were found. In correlation analysis, there was no association found between age, BMI, insulin dose and IMCL or *k*_*CK*_. Skeletal muscle *k*_*CK*_ correlated with hepatic *k*_*ATP*_ over the whole study cohort (r = 0.560, p = 0.020).

### Myocardial lipid stores and heart function

No differences were observed for MYCL and PERI. Parameters of cardiac function were comparable in T1DM and healthy controls (Table 2). No significant correlations with diabetes duration, insulin dose and anthropometric characteristics were found.

**Table 2:** Myocardial lipids (MYCL), pericardial fat mass (PERI) and heart function in T1DM and controls.

	T1DM	Controls	p-value
MYCL (% of w.s.)	0.45 ± 0.11	0.56 ± 0.3	n.s.
PERI (cm^2^)	15 ± 6	22 ± 9	n.s.
Heart rate (bp/min)	66 ± 9	68 ± 15	n.s.
EF (%)	67 ± 5	68 ± 6	n.s.
EDV (ml/m^2^)	55 ± 15	52 ± 11	n.s.
ESV (ml/m^2^)	18 ± 7	16 ± 5	n.s.
SV (ml/m^2^)	37 ± 8	36 ± 8	n.s.
Cardiac index (l/min/m^2^)	2.5 ± 0.6	2.4 ± 0.5	n.s.
Enddiastolic mass (g/m^2^)	64 ± 8	68 ± 11	n.s.
Peak E (ms)	313 ± 82	244 ± 55	n.s.
Peak A (ms)	216 ± 92	192 ± 59	n.s.
E/A ratio	1.66 ± 0.7	1.40 ± 0.5	n.s.

EF: ejection fraction; EDV: enddiastolic volume; ESV: endsystolic volume; SV: stroke volume.

## Discussion

Recent evidence reports alterations in hepatic mitochondrial function in recently diagnosed T1DM with disease duration of about six months. It was suggested that these changes in energy metabolism might precede later diabetes-related liver disease^16^, which is not supported by our present data in patients with long term disease duration. In our group of patients with T1DM hepatic *k*_*ATP*_ is reduced as well. However, this is not linked with hepatic steatosis, since HCL was significantly lower in our population with long standing T1DM (25 ± 11 years) compared to the matched control group. This is in line with previous studies, showing that liver fat content is significantly lower in T1DM compared to non-diabetic controls^9,10^.

The link between lower rates of ATP synthesis rates together with lower HCL stands in sharp contrast to T2DM^13–15^. A possible explanation might be the unphysiological insulin delivery in T1DM. Ferranini *et al*. reported significantly higher insulin concentrations in the splanchnic region compared to peripheral tissue under fasting conditions in non-diabetic healthy subjects^25^. Whereas under physiological conditions pancreatic insulin is delivered directly to the liver, where approximately half of the insulin is extracted by first-pass effects, there is a relative increase in systemic insulin levels and low portal insulin concentrations following subcutaneous injection^26^. This relative lack of insulin in the splanchnic region might explain increased rates of endogenous glucose production during fasting conditions in patients with T1DM, despite higher hepatic insulin sensitivity^9^. Moreover, lower HCL might be explained by the loss of a physiological portal to peripheral insulin ratio in T1DM, as insulin is the main stimulator of de novo lipogenesis and ectopic triglyceride accumulation^27^.

Since insulin strongly stimulates ATP production^28^, basal rates of hepatic energy metabolism might be lower because of a relative lack of insulin in the splanchnic region. On the other hand, hepatic *k*_*ATP*_ might be decreased in connection with lower HCL in T1DM. Recent studies demonstrate lower ATP synthesis rates in the liver of lean, healthy controls compared to patients with NAFLD, reflecting mitochondrial flexibility in early stages of disease by increasing energy turnover in case of excessive substrate availability. This flexibility is subsequently lost, when liver disease progresses, resulting in inadequately reduced mitochondrial ATP turnover, despite higher HCL^14,15^.

With regard to skeletal muscle, similar to previous reports energy turnover is reduced in our patients with T1DM^28,29^. These alterations might be due to changes in circulating glucose and insulin concentrations. Moreover, insulin deficiency by short-term insulin deprivation prompted a decrease in muscle ATP production rates, but also at mitochondrial gene transcription levels^30^. In our cohort blood glucose values and HbA1c were significantly higher in T1DM compared to controls, probably indicating an at least temporary hypoinsulinemia, which could account for observed changes in skeletal muscles *k*_*CK*_.

There are conflicting results on IMCL in T1DM in the literature. Whereas others^28^ and we did not observe significant differences, IMCL was reported to be significantly higher in T1DM in earlier studies, similar to the insulin resistant state and T2DM^31^. These differences are most likely explained by long-term glucose control in investigated groups of patients, since IMCL was higher in T1DM with higher HbA1c, compared to our rather well controlled patient group.

The initiation of insulin therapy in patients with type 2 diabetes mellitus is associated with an acute increase in myocardial lipid stores^32^. However in contrast to this observation, with regard to the heart, neither ectopic or pericardial fat deposition nor cardiac function was different in T1DM. Our data is in line with previous publications of a large cohort of patients with T1DM participating in the diabetes control and complications trial (DCCT), in which cardiac geometry and function was normal^33^. Also, short-term hyperglycemic dysregulation after partial insulin deprivation for 24 hours had no effect on MYCL^34^. Thus, MYCL adapts differently in T1DM compared to insulin sensitive, healthy subjects, in which even in the absence of circulating free fatty acids an elevation in glucose concentrations significantly increased lipid deposition within the myocardium^24^. This could be explained by tracer studies reporting lower rates of glucose uptake into the myocardium under resting conditions in T1DM resembling cardiac insulin resistance. However, during hyperlipidemia and hyperinsulinemia, the heart is still able to adapt adequately to alterations in substrate availability indicating an intact metabolic flexibility^35^. Therefore, typical features of diabetic cardiomyopathy could not be found in T1DM.

This study has several limitations. First of all, the sample size is relatively small. However, according to sample size calculations based on our previous reports, the included number of patients was sufficient to observe assumed differences in hepatic energy metabolism^14^. Also for the assessment of ectopic lipid content in insulin sensitive tissue previous reports of our study group showed significant differences between groups of similar sample size due to the high sensitivity and low inter- and intraobserver variability of high resolution state of the art MRS techniques^23,24^. Moreover, we are not able to prove our hypothesis that the observed differences in hepatic energy metabolism and lipid content are because of a loss of the physiological portal to peripheral insulin ratio in T1DM during subcutaneous insulin therapy, since only peripheral blood samples were drawn at the study days.

Taken together, our results in patients with long standing T1DM show, that HCL is even lower compared to matched controls, despite lower hepatic and skeletal muscle energy metabolism. This might be explained by unphysiological portal to peripheral insulin ratio or by preserved metabolic flexibility. No differences in IMCL, cardiac fat depots and heart function could be found. Our findings are important for patients suffering from T1DM, as well as for treating physicians, since they indicate that NAFLD as well as cardiac steatosis is no clinically relevant long-term consequence of insulin therapy. Therefore, screening for sequelae of long standing T1DM should focus on well-established cardiovascular risk factors like diabetic nephropathy and LDL-cholesterol^5^ to reduce cardiovascular mortality.
